# Trends in Antibiotic Resistance of *Escherichia coli* Strains Isolated from Clinical Samples (2019–2023): A Hospital-Based Retrospective Analysis

**DOI:** 10.3390/pathogens14090927

**Published:** 2025-09-13

**Authors:** Claudia Daniela Goleanu (Vasiloiu), Corneliu Ovidiu Vrancianu, Daria Adelina Goleanu, Monica Marilena Tantu, Ortansa Csutak

**Affiliations:** 1 Microbiology-Immunology Department, Faculty of Biology, University of Bucharest, 050095 Bucharest, Romania; claudiagoleanu@gmail.com; 2 Department of Laboratory Medicine, “Prof. Dr. Constantin Angelescu” Hospital, Aleea Căuzași 49-51, 030167 Bucharest, Romania; 3 Doctoral School, Carol Davila University of Medicine and Pharmacy, Eroii Sanitari 8, District 5, 050474 Bucharest, Romania; 4 The Research Institute of the University of Bucharest—ICUB, Șoseaua Panduri 90, District 5, 050663 Bucharest, Romania; 5 National Institute of Research and Development for Biological Sciences, 296 Splaiul Independentei, District 6, 060031 Bucharest, Romania; 6 Department of General Medicine, Carol Davila University of Medicine and Pharmacy, Eroii Sanitari 8, District 5, 050474 Bucharest, Romania; dariagoleanu@gmail.com; 7 Department of Medical Assistance and Physical Therapy, Pitesti University Center, Târgu din Vale 1, 110040 Pitești, Romania; tantumonica@yahoo.com; 8 Faculty of Science, Physical Education and Informatics, National University of Science and Technology, Politehnica, Splaiul Independenței 313, District 6, 060042 Bucharest, Romania; 9 Department of Genetics, Faculty of Biology, University of Bucharest, 1-3 Aleea Portocalelor, 060101 Bucharest, Romania; ortansa.csutak@bio.unibuc.ro

**Keywords:** *Escherichia coli*, urinary tract infections, antimicrobial resistance, extended-spectrum beta-lactamases, multidrug resistance

## Abstract

Background: Antimicrobial resistance (AMR) is a major public health concern. Urinary tract infections (UTIs) account for up to 85–90% of community-acquired cases. The COVID-19 pandemic disrupted healthcare access and may have influenced resistance patterns. In this context, we retrospectively evaluated the antibiotic resistance dynamics of various bacterial strains isolated between 2019 and 2023 in a hospital unit; Methods: A total of 8217 clinical specimens (urine, wound secretions, sputum, pharyngeal exudate, nasal exudate, tracheal secretions, vaginal and cervical secretions, puncture fluids, purulent secretions, blood, ear secretions, eye secretions) were processed using standard microbiological techniques. Pathogen identification and susceptibility testing were performed with the VITEK 2 Compact system, following CLSI guidelines. Results: Following the analysis of 8217 clinical samples collected over a five-year period (2019–2023), a total of 2900 microorganisms were isolated and identified. Among these, the most frequently encountered were *E. coli* strains, with 1204 isolates. Urine cultures represented 71.3% of all processed samples. Out of these 5860 urine cultures, 1530 (26%) were positive. The resistance of *E. coli* strains to ampicillin (48–55.2%), trimethoprim/sulfamethoxazole (22.9–34%), and ciprofloxacin (21.4–31.5%) remained high throughout the period. ESBL-producing strains peaked at 17.6% in 2020, with multidrug resistance rates ranging from 14% to 22.4%. Conclusions: *E. coli* strains displayed persistently high resistance to ampicillin, trimethoprim/sulfamethoxazole, and ciprofloxacin, with peaks in ESBL production and multidrug resistance during the COVID-19 pandemic. These trends underscore the importance of continuous surveillance and antibiotic stewardship, with direct implications for empirical UTI therapy and broader strategies to mitigate the public health impact of antimicrobial resistance.

## 1. Introduction

Although *Escherichia coli* is part of the normal intestinal microbiota, certain pathogenic strains are a leading cause of clinical infections worldwide [1], particularly urinary tract infections (UTIs) [2], accounting for up to 85–90% of community-acquired cases [3,4,5]. However, less common pathogens such as *Klebsiella pneumoniae*, *Proteus mirabilis*, *Pseudomonas aeruginosa*, *Enterococcus faecalis*, group B *Streptococcus*, *Staphylococcus saprophyticus*, *S. aureus*, *S. epidermidis*, and other bacteria can also cause opportunistic UTIs [6,7,8,9]. According to the Global Burden of Foodborne Diseases report, the incidence of *E. coli* infections is increasing, with approximately 111 million illnesses and 63,000 deaths attributed to diarrheagenic *E. coli* annually worldwide [1]. In clinical settings, UTIs cause different diseases, including asymptomatic/symptomatic bacteriuria, acute, chronic, and recurrent infections, representing a significant challenge in treating UTI patients [10]. This substantial burden necessitates a comprehensive understanding of the pathogen’s impact and its growing antimicrobial resistance crisis.

The COVID-19 pandemic disrupted healthcare access, microbiological diagnostics, and antibiotic prescribing behaviors. These disruptions may have influenced resistance trends, with several studies reporting atypical shifts in antimicrobial resistance patterns during the pandemic [11,12,13,14,15]. In Romania, COVID-19 surveillance began on 27 January 2020, the first case being registered in February 2020. In 2020 and 2021, there were 1,813,823 cases and 58,971 deaths [16]. The pandemic also temporarily reduced AMR surveillance capacity and routine microbiology testing in many healthcare settings, potentially affecting the detection of resistant strains.

At the same time, *E. coli*’s capacity to develop resistance mechanisms, such as extended-spectrum beta-lactamase (ESBL) production, efflux pump overexpression, and porin alterations, has severely limited therapeutic options [17]. While global studies have documented the spread of multidrug-resistant (MDR) and fluoroquinolone-resistant *E. coli* [1,18,19,20,21], national data are more limited and often focused on pediatric populations. In Romania, focused surveillance studies confirm a concerning rise in resistant *E. coli* isolates. A five-year study (2018–2022) reported that 33.3% of urinary *E. coli* isolates were MDR, with 13.9% showing ESBL production and 0.2% producing carbapenemases [22]. Other data from Southeastern Romania revealed significant post-pandemic increases in resistance to fluoroquinolones and third-generation cephalosporins [23], while pediatric data confirm ongoing high resistance to ampicillin, amoxicillin, and norfloxacin [24].

Despite global and pediatric data, there is a scarcity of recent, adult-focused, locally contextualized AMR surveillance for *E. coli* urinary isolates in Romania, particularly regarding trends before and during the COVID-19 pandemic and their implications for empirical treatment. To address this gap, we conducted a retrospective study to evaluate the temporal dynamics (2019–2023) of antibiotic resistance among *E. coli* strains isolated from a hospital unit with medical and surgical wards. By analyzing susceptibility profiles from various clinical specimens (urine, wound secretions, sputum, pharyngeal exudate, nasal exudate, tracheal secretions, vaginal and cervical secretions, puncture fluids, purulent secretions, blood, ear secretions, eye secretions), we aim to inform local antimicrobial stewardship policies, optimize empirical treatment guidelines, and strengthen infection control strategies to limit ESBL-producing and MDR *E. coli* in clinical settings.

## 2. Materials and Methods

### 2.1. Ethical Approval and Data Anonymization

This retrospective, non-interventional study was based solely on anonymized laboratory and microbiological data collected during routine clinical care. No patient identifiers were accessed or used, and no direct contact with patients occurred at any stage of the research. Therefore, in accordance with the General Data Protection Regulation (GDPR) and national ethical guidelines for retrospective studies using anonymized data, informed consent was not required.

### 2.2. Sample Collection

Between 2019 and 2023, a total of 8217 clinical specimens (urine, sputum, wound secretions, cervical secretions, puncture fluids, purulent secretions, pharyngeal exudate, nasal exudate, otic secretion, urethral secretion, tracheal secretion, blood) were collected from patients admitted to the medical, surgical, and ICU wards of the study hospital. Samples were obtained within each ward and transported to the Medical Analysis Laboratory for microbiological analysis.

While a wide range of specimens was processed, *E. coli* isolates were recovered almost exclusively from urine, wound secretions, and sputum. No *E. coli* isolates were obtained from pharyngeal, nasal, or vaginal/cervical secretions, which were included in the overall count of processed samples but not in the *E. coli*-specific analysis.

Only specimens collected from inpatients in the medical, surgical, or ICU wards that yielded *E. coli* isolates subsequently identified and tested for antibiotic susceptibility using the Vitek 2 Compact system (bioMérieux, France, Advanced Expert System software, version 9.01) were included in the resistance analysis. Contaminated samples were excluded when polymicrobial growth without a dominant pathogen or non-significant mixed flora was detected.

### 2.3. Bacterial Isolation

For microbiological analysis, samples were inoculated onto the following culture media: Columbia blood agar with 5% sheep blood, CLED medium, chromogenic medium for urinary infections, and Sabouraud medium with chloramphenicol. Inoculation was performed using sterile disposable loops inside a microbiological safety cabinet. Plates were incubated aerobically at 35–37 °C for 18–24 h. After incubation, plates were examined for the presence of pathogenic bacteria. If no bacterial growth was observed, incubation was extended for an additional 24 h (except for urine samples).

### 2.4. Bacterial Identification

Isolated colonies were used to prepare a homogeneous bacterial suspension with a density of 0.5–0.63 McFarland standard, measured with the Vitek 2 DensiChek device (BioMérieux, Marcy-l’Étoile, France). Suspensions were prepared in sterile saline solution (0.45–0.50% NaCl, pH 4.5–7) in 12 × 75 mm polystyrene tubes. Identification was performed using standard microbiological and biochemical methods, with the automated Vitek 2 Compact system (BioMérieux, Marcy-l’Étoile, France) for both strain identification and antibiotic susceptibility testing. The system was technically verified annually, and external quality control was performed quarterly with consistent results.

For identification of Gram-negative bacilli, Vitek 2 GN cards were used, which include 47 biochemical tests and a negative control well. These cards measure carbon source utilization, enzymatic activity, and resistance markers, generating a biochemical profile based on changes in bacterial growth and metabolism during incubation. Inoculum transfer to the identification and susceptibility cards was performed automatically within the Vitek 2 Compact filling chamber.

### 2.5. Antibiotic Susceptibility Testing

Antibiotic susceptibility testing was performed using the Vitek 2 Compact system (AST N-204 and AST N-222 cards) according to the manufacturer’s instructions. Results were interpreted using CLSI guidelines, which we acknowledge as a limitation given that EUCAST criteria are mandatory in Europe. Extended-spectrum beta-lactamase (ESBL) production was detected phenotypically using the Vitek 2 system. MDR was defined as resistance to three or more antimicrobial classes. The same methodology and antibiotic panel were consistently applied throughout the study (2019–2023).

### 2.6. Statistical Analysis

For the statistical analysis, printed tables were used to manually record data for each identified microorganism tested for antibiotic susceptibility. Each table contained the following columns: sequential number, patient identification code, type of specimen, hospital ward, patient sex, ESBL production, MDR status, and resistance to the antibiotics tested.

A separate table was completed for each study year (2019–2023), containing all *E. coli* strains tested. The data were extracted from the hospital’s electronic medical system, and the results were generated using the Vitek 2 Compact analyzer.

For each antibiotic tested, the number of *E. coli* strains resistant to that antibiotic was counted, and the percentage was calculated relative to the total number of *E. coli* strains isolated in that year. The same procedure was applied to each table’s ESBL and MDR columns.

In addition to descriptive statistics, year-to-year changes in resistance proportions were assessed using the Chi-square test for independence, with a *p*-value < 0.05 considered statistically significant. Statistical analyses were performed using aggregated annual counts for each antibiotic and phenotype (ESBL, MDR) from 2019 to 2023.

### 2.7. Representativeness and Limitations

This study is based on data from a single secondary-care hospital with medical, surgical, and ICU wards, serving a mixed urban and peri-urban population. While the dataset includes many *E. coli* isolates collected over five years and covers diverse clinical specimens, the findings may not fully represent AMR patterns in other Romanian regions or tertiary and primary care settings. Local factors like patient demographics, referral patterns, case mix, and antibiotic prescribing practices may influence resistance rates. Moreover, as a hospital-based study, the data likely overrepresent more severe or complicated infections than community-acquired cases managed in outpatient settings. These factors should be considered when extrapolating the results beyond this institution.

## 3. Results

### 3.1. Distribution of Analyzed Clinical Specimens

**Figure 1:**
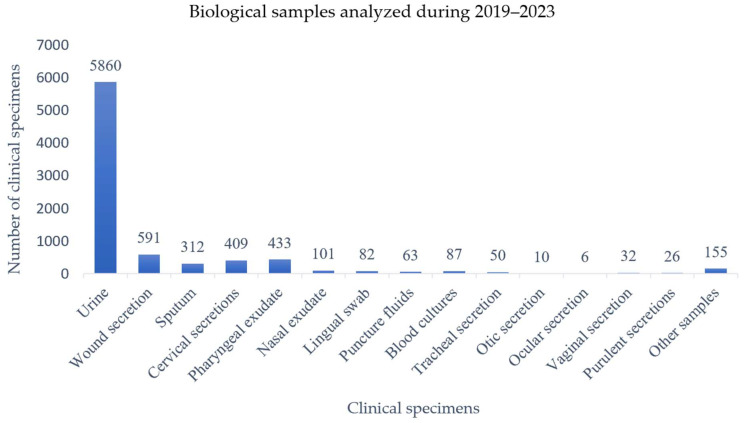
Clinical specimens collected from patients and analyzed during 2019–2023. Wound secretions and purulent secretions represent distinct specimen categories, with the former referring to exudates collected from surgical or traumatic wounds and the latter referring to pus samples obtained from abscesses or localized purulent collections. Less frequent specimen types are also included for completeness: lingual swab (n = 82), tracheal secretion (n = 50), ocular secretion (n = 10), and ear secretion (n = 6).

Between 2019 and 2023, a total of 8217 clinical specimens were collected from patients admitted to the hospital. The majority of these, 5860 (71.3%), consisted of urine samples, indicating the predominance of suspected urinary tract infections in the hospital’s clinical case mix. Wound secretions ranked second, with 591 samples (7.2%), followed by pharyngeal exudates (433; 5.3%), cervical secretions (409; 5%), and sputum samples (312; 3.8%). Less frequently analyzed specimens included nasal exudates (101; 1.2%) and blood cultures (87; 1%). Other clinical specimens, each with frequencies below 1%, are detailed in Table 1 and Figure 1, which also present year-by-year data for all specimen types.

### 3.2. Distribution of Isolated Microorganisms and the Impact of the Pandemic on Laboratory Activity

**Figure 2:**
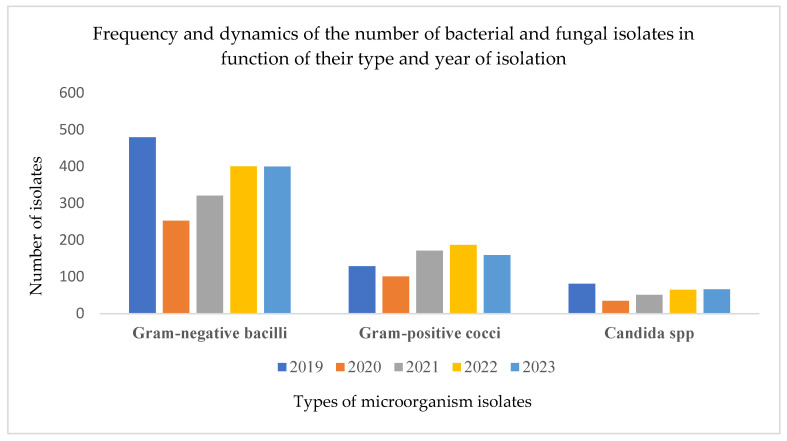
Frequency and dynamics of the number of bacterial and fungal isolates in function of their type and year of isolation. Data were extracted from the hospital’s Laboratory Information System, ensuring comprehensive inclusion of all processed samples.

Over the five-year period from 2019 to 2023, a total of 2900 microorganisms were isolated and identified. These included 1855 strains of Gram-negative bacilli, 747 Gram-positive cocci, and 298 strains of *Candida* spp. A notable decline in isolate numbers was recorded in 2020, coinciding with the onset of the COVID-19 pandemic (11 March 2020–5 May 2023). This reduction was likely due to restricted hospital access, isolation measures, and a temporary drop in routine sample collection. Specifically, the number of isolates dropped from 690 in 2019 to 389 in 2020, a decrease of nearly 44%, highlighting the indirect impact of the pandemic on diagnostic microbiology activity. Among the Gram-negative bacilli, *E. coli* was the most frequently identified species and is the primary focus of the present analysis (Figure 2).

### 3.3. Urine Cultures and Infection Rates

Between 2019 and 2023, a total of 5860 urine samples were analyzed, with 1530 cultures testing positive for urinary tract infections, yielding an overall positivity rate of 26%. While the absolute number of urine cultures decreased significantly in 2020 due to the onset of the COVID-19 pandemic, the proportion of positive results remained relatively stable across the five years, ranging from 22% to 30% (Table 2). The slight peak in positivity during 2020 may reflect a shift toward testing more symptomatic or high-risk individuals under restrictive hospital access protocols.

### 3.4. E. coli Prevalence

**Figure 3:**
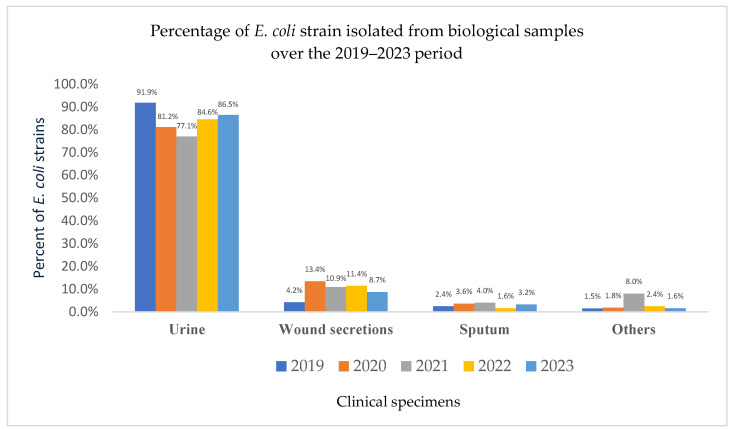
Percentage of *E. coli* strains isolated from clinical specimens over the 2019–2023 period. While the study primarily focused on urinary tract infections, non-urine specimens (e.g., wound secretions, sputum, and others) are shown for epidemiological context, reflecting the broader distribution of *E. coli* across different infection sites.

During the study period (2019–2023), a total of 8217 clinical specimens were processed, and 2900 microorganisms were isolated and identified. Among these, *E. coli* was the most frequently identified microorganism, with a total of 1204 isolates. In 2019, 332 *E. coli* strains were recorded. This number dropped sharply to 165 in 2020—a 50% decrease coinciding with the onset of the COVID-19 pandemic. A gradual recovery was observed in the following years, with 201 strains identified in 2021, and further increases to 254 in 2022 and 252 in 2023 (Figure 3). Although the analysis primarily focused on urinary tract infections, *E. coli* prevalence in other clinical specimens (e.g., wound secretions, sputum) was also reported to provide epidemiological context and to illustrate the bacterium’s broader role in both community-acquired and healthcare-associated infections.

### 3.5. Source and Demographic Distribution of E. coli Isolates

Over the five-year period, the majority of *E. coli* isolates originated from urine samples (85.3%), followed by wound secretions (9%) and sputum (2.8%). An analysis by sex, focusing on the three most commonly analyzed specimen types between 2019 and 2023, showed that 82.8% of the *E. coli* strains were isolated from female patients, while only 17.2% were from male patients.

### 3.6. Five-Year Surveillance of Antibiotic Resistance Patterns in E. coli Strains

**Figure 4:**
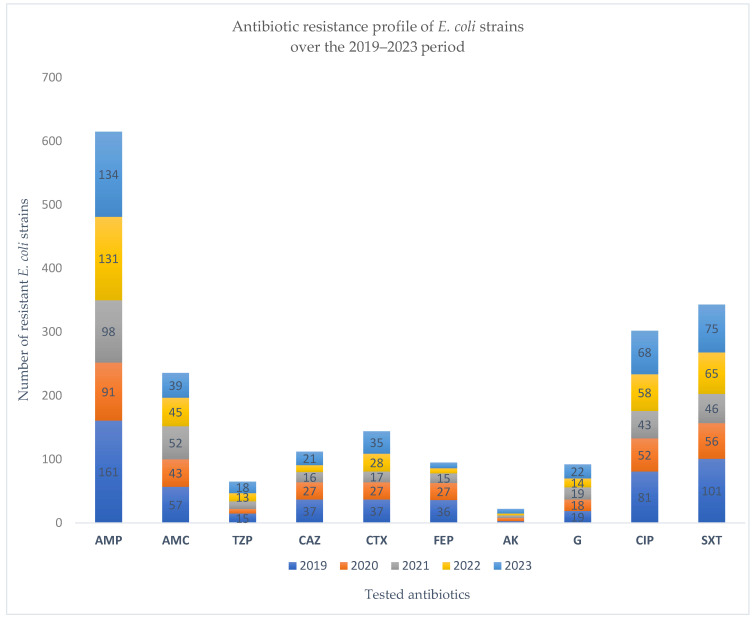
Antibiotic resistance profiles of *E. coli* strains over the 2019–2023 period (absolute counts).

**Figure 5:**
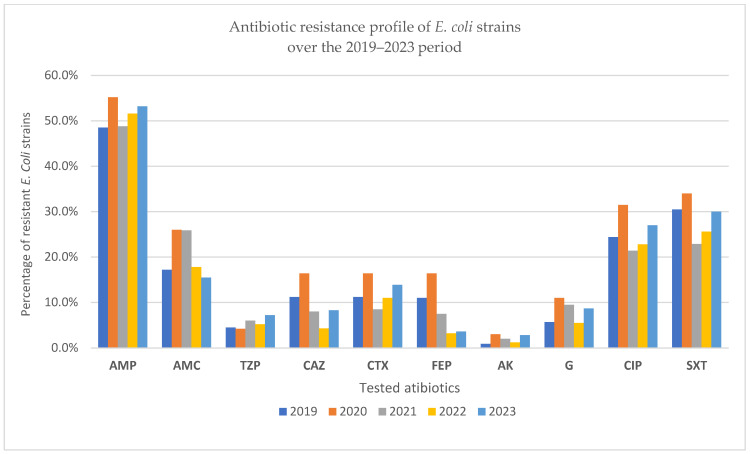
Antibiotic resistance profiles of *E. coli* strains over the 2019–2023 period (percentages).

In 2019, nearly half (48.5%) of the *E. coli* strains isolated were resistant to ampicillin, reaffirming its limited therapeutic value. Resistance to amoxicillin/clavulanic acid was notably lower (17.2%), and piperacillin/tazobactam remained largely effective (4.5% resistance). Resistance to third-generation cephalosporins (ceftazidime, cefotaxime, cefepime) was moderate (~11%), while aminoglycosides, particularly amikacin, showed excellent activity. In contrast, resistance to ciprofloxacin (24.4%) and trimethoprim/sulfamethoxazole (30.5%) was substantial, limiting their empirical use. ESBL-producing strains accounted for 13%, and 16.9% were MDR, underscoring the growing challenge of treating *E. coli* infections with first-line antimicrobials (Figure 4 and Figure 5).

In 2020, ampicillin resistance among *E. coli* isolates rose to over 55%, confirming the persistent inefficacy of this agent. Resistance to third-generation cephalosporins and amoxicillin/clavulanic acid increased compared to 2019, suggesting a pandemic-related shift in prescribing patterns. Ciprofloxacin and trimethoprim/sulfamethoxazole resistance also remained high, exceeding 30%, further limiting oral therapeutic options. Notably, the rate of ESBL-producing strains peaked at 17.6%, and MDR reached 22.4%, both the highest recorded across the five-year period, highlighting the potential impact of COVID-19-related disruptions on resistance dynamics (Figure 4 and Figure 5).

**Figure 6:**
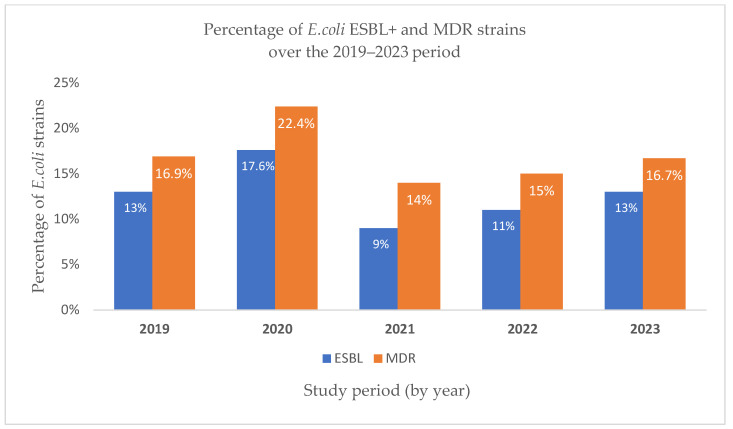
Percentage of *E. coli* ESBL+ and MDR strains over the 2019–2023 period.

In 2021, ampicillin resistance among *E. coli* strains remained high (nearly 49%), sustaining its role as a weak therapeutic option. While resistance rates for third-generation cephalosporins slightly declined compared to 2020, fluoroquinolone and trimethoprim/sulfamethoxazole resistance persisted above 20%, underscoring limited oral treatment alternatives. Notably, the proportion of ESBL-producing strains decreased by almost half from the previous year, and MDR dropped to 14%, suggesting a possible transient improvement in antimicrobial pressure post-2020. These findings indicate partial stabilization in resistance patterns following the initial pandemic disruptions (Figure 6).

In 2022, *E. coli* resistance levels stabilized compared to prior years. Ampicillin resistance remained high (51.6%), consistent with its limited clinical utility. Third-generation cephalosporin resistance, especially cefotaxime (11%), was slightly elevated from 2021, possibly reflecting a rebound in healthcare-associated exposure. Resistance to ciprofloxacin (22.8%) and trimethoprim/sulfamethoxazole (25.6%) also persisted, narrowing oral treatment options. Notably, ESBL production increased slightly compared to 2021, while MDR was reported in nearly 15% of isolates, suggesting a moderate but steady resistance pressure post-pandemic (Figure 6).

In 2023, *E. coli* resistance patterns remained broadly consistent with the previous year, with ampicillin resistance persisting above 50%, underscoring its continued ineffectiveness as an empirical choice. Third-generation cephalosporin resistance remained moderate, with cefotaxime resistance (13.9%) slightly elevated compared to 2022, possibly reflecting residual post-pandemic selection pressures. Ciprofloxacin and TMP/SMX resistance rates reached 27% and 30%, respectively, the highest values since 2019, highlighting an ongoing decline in oral therapeutic options (Figure 4 and Figure 5). ESBL production stabilized at 13%, while MDR prevalence reached 16.7%, indicating a steady presence of high-risk, resistant strains despite the return to pre-pandemic clinical activity (Figure 6).

Analyzing the table below, which presents the percentage of antibiotic resistance for *E. coli* strains isolated during the period 2019–2023, it is observed that the highest resistance was consistently recorded for Ampicillin. This is followed by resistance to Trimethoprim/Sulfamethoxazole, Ciprofloxacin, and Amoxicillin/Clavulanic acid. These four antibiotics showed the highest percentages of resistance over the five years compared to the other antibiotics tested (Table 3).

Comparing the number of *E. coli* strains producing extended-spectrum beta-lactamases (ESBL+) over the five years analyzed, the highest percentage was recorded in 2020 (17.6%). The percentages in 2019 and 2023 were identical, both at 13%. In 2022, the proportion of ESBL-producing *E. coli* strains was 11%, while the lowest percentage was observed in 2021, at 9%, as shown in Table 4.

Regarding multidrug-resistant (MDR) *E. coli* strains, a similar ranking is noted: 2020 had the highest proportion (22.4%), followed by 2019 and 2023 with closely aligned values (16.9% and 16.7%, respectively). In 2022, the percentage was 15%, and the lowest MDR rate was again observed in 2021, at 14%.

Statistical comparison of resistance rates over the study period showed significant year-to-year variation for ampicillin (*p* < 0.001), ciprofloxacin (*p* = 0.02), and trimethoprim/sulfamethoxazole (*p* = 0.01). ESBL prevalence also varied significantly between years (*p* = 0.04), peaking in 2020, while MDR rates showed a moderate but non-significant fluctuation (*p* = 0.07). These results support that several of the observed changes in resistance profiles over time were unlikely due to random variation alone.

## 4. Discussion

This five-year analysis of 1204 *E. coli* isolates from 8217 clinical specimens provides an overview of local antimicrobial resistance trends in a secondary-care hospital. Most isolates originated from urine cultures (91.3%), followed by wound secretions and sputum. Ampicillin resistance remained the highest across all years (>48%), followed by trimethoprim/sulfamethoxazole (22.9–33.9%) and ciprofloxacin (21.4–31.5%), while resistance to nitrofurantoin and fosfomycin was consistently low. ESBL prevalence ranged from 9.0% to 17.6%, peaked in 2020, and MDR rates ranged between 14.0% and 22.4%. The total number of *E. coli* isolates declined sharply in 2020 (−50% compared to 2019) before gradually increasing in subsequent years.

The analysis of *E. coli* isolates collected between 2019 and 2023 reveals several significant AMR trends, with yearly fluctuations shaped by biological and external factors, including the COVID-19 pandemic. Ampicillin resistance remained consistently high, exceeding 48% in all years.

This finding aligns with data from pediatric cohorts in Romania, which reported high resistance rates to ampicillin among *E. coli* strains in children with UTIs [23,24]. Similarly, a 2022 cross-sectional study covering clinical and environmental samples reported ampicillin resistance in 83.4% of clinical *E. coli* isolates, highlighting a broad and persistent resistance problem in adult and neonatal populations [20]. Comparable levels were also noted in Indonesia (60%) and Iraq (89.8%), suggesting that ampicillin resistance is not only consistent but geographically widespread across clinical populations [25,26].

Resistance rates to ciprofloxacin and trimethoprim/sulfamethoxazole in our adult population were notably higher than those typically reported in pediatric populations, underscoring potential age-related differences in antibiotic exposure or prescribing practices. For instance, ciprofloxacin resistance in our study reached 27%, compared to only 1.4% among children with UTIs in a Greek study by Vazouras et al. (2020) [27]. Similarly, TMP/SMX resistance was 30% in our cohort versus 26.5% in the pediatric population, suggesting it remains a suboptimal empirical option across age groups. Global pediatric studies have reported even higher TMP/SMX resistance, such as rates exceeding 89% in neonatal isolates and 50% in Indonesia [21], reinforcing concerns about its waning clinical utility.

Third-generation cephalosporin resistance was variable in our setting (4–16%), while remaining much lower in pediatric reports, only 1.7% in the study by Vazouras et al. [27]. However, an earlier Romanian study [28] showed a much higher ESBL+ rate (80.9% for *E. coli*), indicating strong regional and institutional variability. In our hospital, ESBL+ rates peaked at 17.6% in 2020 and remained between 9% and 13% in other years. Such discrepancies may reflect differences in population susceptibility, previous antibiotic exposure, and referral bias (e.g., nephrology centers with higher anomaly prevalence). Supporting this, a recent neonatal study identified ESBL-producing *E. coli* in 23% of screened neonates and found that 5% of maternal hands and 22% of neonatal cots were contaminated with ESBL+ strains. Ampicillin and TMP/SMX resistance in these isolates reached 100% and 89%, respectively [21]. Additionally, in another recent study, the MDR rate in *E. coli* reached 63% with 32% classified as XDR, and third-generation cephalosporin resistance was notably high in *K. pneumoniae* (71%), findings aligned with the global expansion of resistance genes [29].

Our adult population had the most frequent AMR in 2020 (22.4%). In pediatric settings, MDR risk factors such as long-term prophylaxis and urinary tract anomalies have also been identified, echoing our findings where patients with comorbidities often harbor MDR strains.

Interestingly, in the pediatric cohort, ESBL+ infections were paradoxically more frequent in children without prior anomalies or prophylaxis, contradicting earlier assumptions and suggesting that community acquisition of resistant strains may be emerging even in younger populations. Supporting this trend, a retrospective study in Brazil revealed that recent hospitalization significantly predicted MDR infections, particularly with Gram-negative fermenting bacilli [30]. In contrast, a study in Iraq found a notably higher MDR prevalence (57.3%) than ours, with *E. coli* MDR rates reaching 46%, underscoring the geographic disparities and stressing the importance of local surveillance [26].

The peak in AMR, ESBL production (17.6%), and MDR rates (22.4%) observed in our study during 2020 coincided with the onset of the COVID-19 pandemic, suggesting that pandemic-related disruptions, such as reduced hospital admissions, restricted diagnostic activity, and empirical antibiotic prescribing, may have influenced resistance dynamics.

Statistical comparison of resistance rates over the study period showed significant year-to-year variation for ampicillin (*p* < 0.001), ciprofloxacin (*p* = 0.02), and trimethoprim/sulfamethoxazole (*p* = 0.01). ESBL prevalence also varied significantly between years (*p* = 0.04), peaking in 2020, while MDR rates showed a moderate but non-significant fluctuation (*p* = 0.07). These results support that several of the observed changes in resistance profiles over time were unlikely due to random variation alone.

Our findings align with those reported by Fong Coronado et al. [11], who documented an increase in β-lactam resistance in *E. coli* and *K. pneumoniae* during the pandemic in Mexico. Similarly, Golli et al. [15] observed a significant rise in colistin and tigecycline resistance in *Acinetobacter* spp. and *Klebsiella* spp. after the pandemic, but also noted a statistically significant decrease in resistance among *E. coli* isolates to ceftazidime and aminoglycosides.

In Finland, Ilmavirta et al. [13] reported a 23.3% reduction in *E. coli* urine isolates tested during 2020–2021. However, they observed a decline in ESBL-producing *E. coli* strains across urine and blood cultures, especially in older adults. This finding contrasts with our data, where the ESBL rate peaked in 2020, potentially reflecting localized differences in antibiotic use or infection control practices.

Additional evidence comes from Stanley et al. [12], who described abrupt shifts in bloodstream infection resistance rates in the UK, with immediate declines in ciprofloxacin and piperacillin-tazobactam resistance following lockdowns, suggesting that altered transmission patterns and reduced person-to-person contact influenced AMR trends. Collignon et al. [14] found that international travel restrictions and decreased population mobility had a more pronounced effect on resistance rates than on antibiotic use itself, an effect likely contributing to resistance suppression in some settings. Together, these comparisons emphasize that while the pandemic globally disrupted AMR dynamics, local responses, healthcare access, and infection control practices strongly shaped the direction and magnitude of resistance trends.

From a therapeutic perspective, our local trends thus complement those Raya and collaborators observed, validating the continued efficacy of non-β-lactam options such as nitrofurantoin and fosfomycin, especially where β-lactam resistance is increasing [31]. Kasanga et al. [20] further support this, where fosfomycin and nitrofurantoin remained among the few antibiotics with low resistance in clinical *E. coli* strains (3% and 2%, respectively). Notably, while ciprofloxacin remained the most effective agent in a recent multicenter study in diabetic and non-diabetic female patients [32], our local resistance rate of 27% in 2023 may limit its empirical utility, particularly in high-risk populations.

Finally, interpretation of susceptibility results must also consider methodological variability. A recent study comparing automated systems (PHOENIX BD™ and VITEK2) with the disk diffusion method reported significant discrepancies, particularly when different interpretive standards (CLSI vs. BrCAST) were applied [33]. In our study, VITEK2 was consistently used and interpreted via CLSI guidelines, yet awareness of such limitations remains crucial for clinical accuracy.

The findings have direct implications for local clinical practice and policy. The persistently high resistance to ampicillin, trimethoprim/sulfamethoxazole, and ciprofloxacin suggests these agents should be avoided for empirical treatment of uncomplicated UTIs in this setting. Instead, the sustained activity of nitrofurantoin and fosfomycin supports their continued inclusion as first-line options in local treatment guidelines. Regular updates to empiric therapy protocols, informed by ongoing surveillance, are essential to maintain effectiveness. At the policy level, strengthening antimicrobial stewardship programs and ensuring the availability of effective oral agents in both hospital and community settings should be prioritized. Future research should include multi-center studies with molecular characterization of resistance mechanisms to better understand the genetic drivers of resistance and to track the emergence of high-risk clones.

This study has several limitations that should be considered when interpreting the results. The analysis was conducted in a single secondary-care hospital, which may limit the generalizability of the findings to other regions or healthcare settings. Although measures were taken to include only the first *E. coli* isolate per patient per specimen type and year, the possibility of repeated sampling from the same individuals cannot be completely excluded. In addition, no genotypic characterization of resistance determinants was performed, which restricts the ability to link phenotypic patterns to specific genetic mechanisms. Future multi-center studies should incorporate prospective isolate storage and molecular analyses to provide a broader and more detailed understanding of AMR trends and to track the emergence of high-risk MDR and ESBL-producing *E. coli* clones.

The study reveals persistently high levels of antibiotic resistance in *E. coli*, with only moderate variations over the analyzed period. The findings underscore the ongoing need for active microbiological surveillance, regular updates to therapeutic guidelines, and the implementation of rigorous antibiotic stewardship measures tailored to the local context of the hospital.

## 5. Conclusions

This study highlights consistently high resistance rates in *E. coli* isolates collected between 2019 and 2023, particularly to ampicillin, TMP/SMX, and ciprofloxacin, with marked fluctuations during the COVID-19 pandemic. Our findings align with national and international data and reveal substantial regional variability in ESBL+ and MDR prevalence, emphasizing the influence of patient age, comorbidities, and recent healthcare exposure on resistance patterns.

For clinicians, these findings support avoiding empirical use of ampicillin, trimethoprim/sulfamethoxazole, and fluoroquinolones for uncomplicated urinary tract infections in this setting. Nitrofurantoin and fosfomycin, which maintain low resistance rates, should remain first-line options, and susceptibility testing should be prioritized in high-risk patients, especially those with comorbidities or suspected MDR/ESBL infections.

For microbiologists, maintaining consistent testing methodologies and providing timely, detailed susceptibility reports will help guide targeted therapy and monitor resistance trends.

For policymakers, strengthening local AMR surveillance, ensuring the availability of effective oral agents in both hospital and community settings, and supporting antimicrobial stewardship programs tailored to local resistance data are essential to preserve treatment effectiveness.

This study is limited by its single-center design, potential repeated sampling, and the absence of genotypic characterization of resistance determinants, which may restrict the generalizability and mechanistic interpretation of the findings.

## Figures and Tables

**Table 1:** Clinical specimens collected from patients and analyzed during 2019–2023.

Year	Urine	Wound Secretions	Sputum	Pharyngeal Exudate	Nasal Exudate	Tracheal Secretion	Otic Secretion	Vaginal Secretion	Cervical Secretion	Puncture Fluid	Blood Culture	Lingual Swab	Ocular Secretion	Purulent Secretion	Other Products *	Total
2019	1542	87	79	126	18	8	7	5	71	18	14	28	-	6	14	2023 (24.6%)
2020	627	89	38	45	10	9	-	14	62	17	16	14	-	5	11	957 (11.6%)
2021	988	131	62	61	22	3	-	1	124	8	26	14	1	3	42	1486 (18.1%)
2022	1183	153	50	86	26	10	2	6	97	10	18	16	3	5	42	1707 (20.7%)
2023	1520	131	83	115	25	20	1	6	55	10	13	10	2	7	46	2044 (24.8%)
Total	5860	591	312	433	101	50	10	32	409	63	87	82	6	26	155	8217

* Other products: urethral swab, bronchoscopic sample, axillary colonization sample, inguinal colonization sample, anal colonization sample.

**Table 2:** Frequency of urinary tract infections in urine samples analyzed during 2019–2023.

Year	Urine Samples	Positive Cultures	% Positive Cultures
2019	1542	443	28.7%
2020	627	188	30%
2021	988	247	25%
2022	1183	321	27%
2023	1520	331	22%
Total 2019–2023	5860	1530	26%

**Table 3:** Antibiotic resistance of *E. coli* strains identified between 2019 and 2023.

Year	AMP	AMC	TZP	CAZ	CTX	FEP	AK	G	CIP	SXT
2019	161 48.5%	57 17.2%	15 4.5%	37 11.2%	37 11.2%	36 11%	3 0.9%	19 5.7%	81 24.4%	101 30.5%
2020	91 55.2%	43 26%	7 4.2%	27 16.4%	27 16.4%	27 16.4%	5 3%	18 11%	52 31.5%	56 34%
2021	98 48.8%	52 25.9%	12 6%	16 8%	17 8.5%	15 7.5%	4 2%	19 9.5%	43 21.4%	46 22.9%
2022	131 51.6%	45 17.8%	13 5.2%	11 4.3%	28 11%	8 3.2%	3 1.2%	14 5.5%	58 22.8%	65 25.6%
2023	134 53.2%	39 15.5%	18 7.2%	21 8.3%	35 13.9%	9 3.6%	7 2.8%	22 8.7%	68 27%	75 30%

AMP = Ampicillin, AMC = Amoxicillin-clavulanic acid, TZP = Piperacillin-Tazobactam, CAZ = Ceftazidime, CTX = Cefotaxime, FEP = Cefepime, AK = Amikacin, G = Gentamicin, CIP = Ciprofloxacin, SXT = Trimethoprim-sulfamethoxazole.

**Table 4:** Variation in ESBL-producing and multidrug-resistant *E. coli* strains during the period 2019–2023.

Year	2019	2020	2021	2022	2023
ESBL	43 13%	29 17.6%	18 9%	28 11%	33 13%
MDR	56 16.9%	37 22.4%	28 14%	38 15%	42 16.7%
Total *E. coli* strains isolated	332	165	201	254	252

Percentages refer to proportions of total isolates per year.

## Data Availability

The original contributions presented in this study are included in the article. Further inquiries can be directed to the corresponding author.
